# Trichosanthis Pericarpium Aqueous Extract Protects H9c2 Cardiomyocytes from Hypoxia/Reoxygenation Injury by Regulating PI3K/Akt/NO Pathway

**DOI:** 10.3390/molecules23102409

**Published:** 2018-09-20

**Authors:** Donghai Chu, Zhenqiu Zhang

**Affiliations:** 1 Liaoning University of Traditional Chinese Medicine, No. 77 Shengming 1st Road, Dalian 11600, Liaoning, China; chubotany@sina.cn; 2 Liaoning Institute of Science and Technology, No. 76 Xianghuai Road, Benxi 117004, Liaoning, China

**Keywords:** trichosanthis pericarpium, hypoxia/reoxygenation, apoptosis, PI3K/AKT/NO

## Abstract

Trichosanthis Pericarpium (TP) is a traditional Chinese medicine for treating cardiovascular diseases. In this study, we investigated the effects of TP aqueous extract (TPAE) on hypoxia/reoxygenation (H/R) induced injury in H9c2 cardiomyocytes and explored the underlying mechanisms. H9c2 cells were cultured under the hypoxia condition induced by sodium hydrosulfite for 30 min and reoxygenated for 4 h. Cell viability was measured by MTT assay. The amounts of LDH, NO, eNOS, and iNOS were tested by ELISA kits. Apoptotic rate was detected by Annexin V-FITC/PI staining. QRT-PCR was performed to analyze the relative mRNA expression of Akt, Bcl-2, Bax, eNOS, and iNOS. Western blotting was used to detect the expression of key members in the PI3K/Akt pathway. Results showed that the pretreatment of TPAE remarkably enhanced cell viability and decreased apoptosis induced by H/R. Moreover, TPAE decreased the release of LDH and expression of iNOS. In addition, TPAE increased NO production and Bcl-2/Bax ratio. Furthermore, the mRNA and protein expression of p-Akt and eNOS were activated by TPAE pretreatment. On the contrary, a specific inhibitor of PI3K, LY294002 not only inhibited TPAE-induced p-Akt/eNOS upregulation but alleviated its anti-apoptotic effects. In conclusion, results indicated that TPAE protected against H/R injury in cardiomyocytes, which consequently activated the PI3K/Akt/NO signaling pathway.

## 1. Introduction

Ischemic heart disease, which includes coronary heart disease (CHD), is one of the leading causes for death all over the world [1,2,3,4]. Angina pectoris is the fatal subtype of CHD, resulting from myocardial ischemia and hypoxia [5]. Currently, the effective therapy for relieving ischemia is early myocardial reperfusion or primary percutaneous coronary intervention (PCI) [6,7]. However, both of them may self-contradictorily lead to further injury, which is known as myocardial ischemia/reperfusion injury (MI/RI) [7,8]. Recently, Chinese herbal medicines, especially combined with herbal formulation, have been widely applied to relieve MI/RI [8,9,10,11].

Trichosanthes Pericarpium, a well-known medicinal herb, is derived from the dried mature pericarp of *Trichosanthes kirilowii* Maxiam or *Trichosanthes rosthornii* Harms [12,13]. It has been reported that TP protects from ischemia reperfusion injury, hypoxia, angina and various cardiovascular diseases in China [4,5,14]. Many studies have verified that TP displayed its protective effect on MI/RI [5,13,14,15]. However, the mechanism by which TPAE affects the above diseases remains unknown, which makes it difficult to apply the efficacy of TP in clinic [16].

Phosphatidylinositol 3-kinase (PI3K)-protein kinase B (Akt) signaling pathway, one of the most important reperfusion injury salvage kinase (RISK) pathways, can suppress cardiomyocyte apoptosis and promote cell survival in H/R cardiomyocytes [17,18]. PI3K-Akt also activates or inhibits downstream target proteins, including eNOS, iNOS, Bcl-2, and Bax via phosphorylation. 

Nitric oxide (NO), generated by the eNOS and iNOS has been implicated in many hypoxia diseases [19,20,21,22,23,24,25]. It has been shown that NO dependent blunting of the hypercontractile response of the heart reduces myocardial injury during the early reperfusion period in a mouse model [26,27]. Although augmented levels of peroxynitrite are associated with induction of cytokines and subsequent impairments in cardiac function, there is also a body of evidence to indicate that physiologically relevant concentrations of peroxynitrite can exert cardioprotective effects during MI/RI [26,28,29,30,31]. NO can also reduce oxidative stress and exert cardioprotection [32].

Thus, in the present study, we evaluated the potential molecular mechanisms of TPAE against H/R induced cardiomyocytes injury via production of NO, and explored that TPAE protected H9c2 cells from H/R injury through activation of the PI3K-Akt signaling pathway by upregulating p-Akt, eNOS, and the ratio of Bcl-2 to Bax and downregulating iNOS. 

## 2. Results

### 2.1. TPAE Inhibited Cell Death Induced by H/R

**Figure 1:**
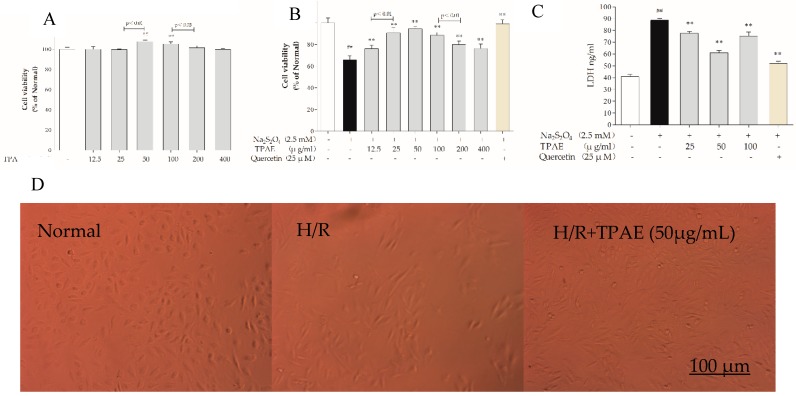
Effects of TPAE on H/R induced H9c2 cell death and morphological changes. H9c2 cells were pretreated with various concentrations of TPAE for 24 h followed by 30 min of hypoxia and 4 h of reoxygenation or only pretreated with various concentrations of TPAE for 24 h. (**A**) Cytotoxicity was detected by MTT assay; (**B**) Cell viability was determined by MTT assay; (**C**) LDH release was measured by ELISA kit; (**D**) Morphology alterations after H/R injury under an inverted phase microscope. Values were represented as mean ± SD (n = 6 for MTT, n = 8 for ELISA, each group). The results were representative of three independent experiments. ^#^ *p* < 0.05, ^##^ *p* < 0.05 vs. normal group; * *p* < 0.05, ** *p* < 0.01 vs. H/R group. Scale bar, 100 μm.

H9c2 cells were incubated with various concentrations of TPAE (12.5, 25, 50, 100, 200, 400 μg/mL) for 24 h and cell viabilities were measured by MTT assay. The results revealed that cell viabilities did not decrease among TPAE-treated groups compared with the normal group. Interestingly, the cell viabilities increased significantly in the 50 and 100 μg/mL TPAE-treated groups, by 7.78% and 5.34%, respectively (107.78% ± 1.38% vs. 100.00% ± 2.02%, *p* < 0.01; 105.34% ± 2.14% vs. 100.00% ± 2.02%, *p* < 0.01, Figure 1A). 

Sodium hydrosulfite (Na_2_S_2_O_4_), a potent reducing agent and oxygen scavenger, is able to instantly lower the partial pressure of oxygen (PO_2_) of the solutions [33]. H9c2 cells were treated with various concentrations of Na_2_S_2_O_4_ (625 μM, 1, 1.25, 2.5, 5 mM) for a fixed time (4 h) or for different lengths of time (10 min, 20 min, 30 min, 40 min or 1 h) at a fixed concentration (2.5 mM) in high glucose DMEM. Based on the previous results, we found that hypoxia/reoxygenation induced by Na_2_S_2_O_4_ decreased cell viability in a concentration-dependent manner [34,35,36,37]. Thereby, a treatment of 2.5 mM Na_2_S_2_O_4_ for 30 min and replacing the normal media for 4 h was applied to induce H9c2 cardiomyocytes H/R injury in subsequent experiments. When H/R was subjected to H9c2 cells, the cell viability decreased significantly (*p* < 0.01); however, pretreatment of TPAE and Quercetin (positive group) could attenuate the injury of H/R on H9c2 cells (Figure 1B). 

Especially cells-treated with TPAE (25, 50 and 100 μg/mL) showed higher viability than other concentrations of TPAE (*p* < 0.01). Therefore, three dosages of TPAE (25, 50 and 100 μg/mL) were chosen as low, middle and high dosages for further experiments. These results were further confirmed by LDH release assay. H/R induced a notable increase of LDH release in H9c2 cells, while TPAE pretreatment effectively attenuated cell injury, with a reduction of 12.44%, 31.12% and 14.97% compared with the H/R group, respectively (Figure 1C). 

Furthermore, the morphological changes of H9c2 cells were observed after H/R treatment. The H9c2 cells in the normal group were fusiform with a full cytoplasm and clear edges (Figure 1D). Differently, the H/R model group showed cell shrinkage and cell fragmentation with inconspicuous edges; this phenotype could be rescued by TPAE (50 μg/mL) (Figure 1D). Therefore, the results showed that TPAE not only had no toxicity on H9c2 cells but improved cell viability and prevented cell damage in H/R-induced H9c2 cells. 

### 2.2. TPAE Prevented Apoptosis in H9c2 Cells after H/R

**Figure 2:**
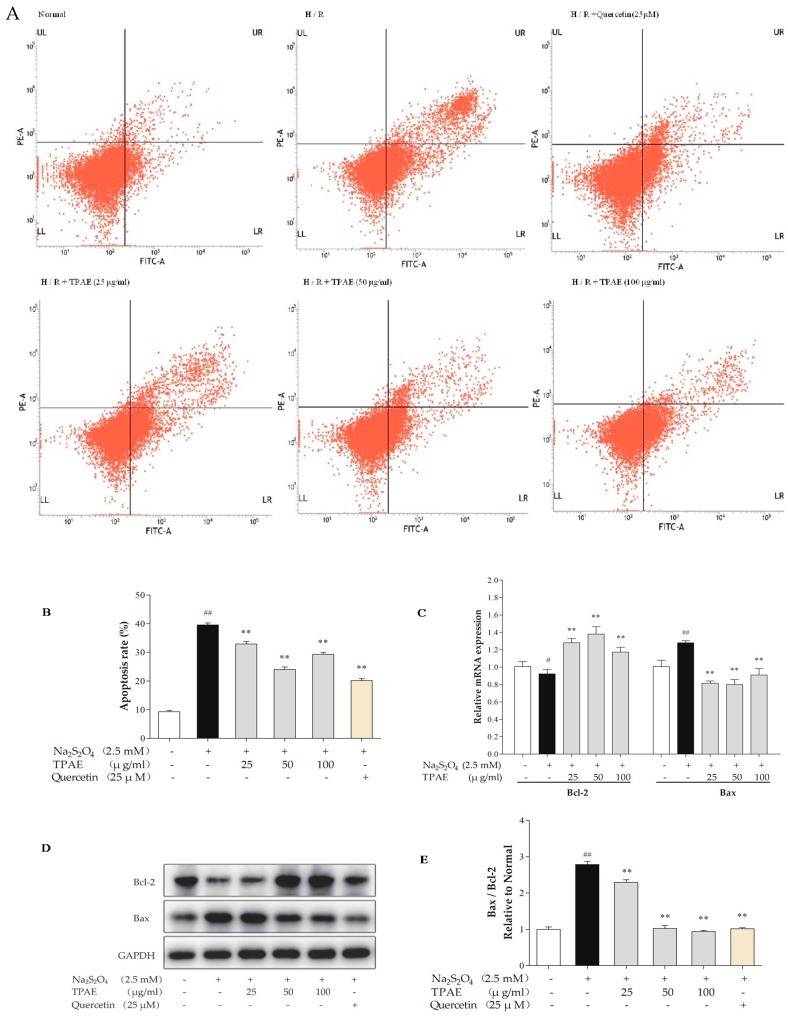
Effects of TPAE on H/R induced H9c2 cardiomyocytes apoptosis. H9c2 cells were pretreated with three dosages of TPAE for 24 h followed by H/R. (**A**) The apoptotic ratio of H9c2 cells was measured by flow cytometry using Annexin V-FITC and PI staining; (**B**) The apoptosis rate was quantified by BD FACSVerse software; (**C**) The relative mRNA expression of Bcl-2 and Bax was determined by qRT-PCR; (**D**) Immunoblots of Bcl-2, Bax and glyceraldehydes-3-phosphate dehydrogenase (GAPDH) was detected by western blotting; (**E**) Quantitative analysis of the ratio of Bax to Bcl-2 in protein expression was evaluated. Values were represented as mean ± SD (n = 3, each group). The results were representative of three independent experiments. ^#^ *p* < 0.05, ^##^ *p* < 0.05 vs. normal group; * *p* < 0.05, ** *p* < 0.01 vs. H/R group.

Myocardial apoptosis contributes to one of the pathological mechanisms of MI/RI [38]. Therefore, we performed Annexin V-conjugated FITC and PI staining to examine the anti-apoptotic effect of TPAE in H9c2 cells after H/R. The results showed that the apoptotic index in the H/R injury group was significantly higher than that in the normal group (39.57% ± 0.69% vs. 9.29% ±0.39%, respectively, *p* < 0.01, Figure 2A,B). However, TPAE (50 μg/mL) pretreatment prior to H/R reduced the apoptosis rate compared to that of cells exposed to H/R (24.04% ± 0.87% vs. 39.57% ± 0.69%, respectively, *p* < 0.01, Figure 2A,B), indicating that TPAE could exert cardioprotective effects by inhibiting apoptosis of H9c2 cells. Besides, the apoptosis markers, Bax and Bcl-2 involved in the execution-phase of cell apoptosis were examined by qRT-PCR and Western blot analyses (Figure 2C–E). Pretreatment with 50 μg/mL TPAE decreased the expression of Bax and enhanced that of Bcl-2, and thus significantly lowered the Bax/Bcl-2 ratio in H/R induced cardiomyocytes (*p* < 0.01, Figure 2E). Thus, the results suggested that TPAE pretreatment could protect H9c2 cells from H/R-induced apoptosis.

### 2.3. TPAE Activated PI3K/Akt Signaling Pathway 

**Figure 3:**
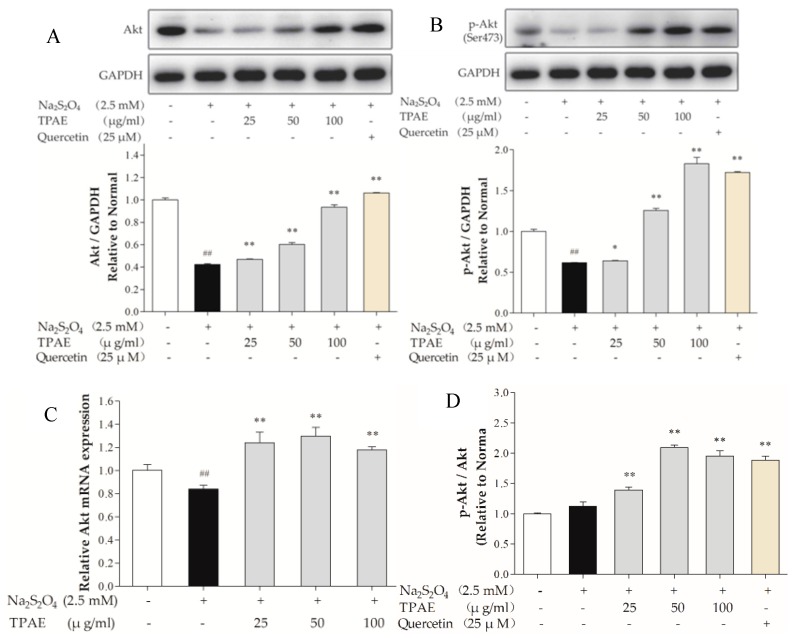
Effects of TPAE on PI3K/Akt signaling pathway. The protein expression of Akt (**A**), p-Akt (Ser473) (**B**), and GAPDH was measured by western blotting. Quantitative analyses of Akt normalized to GAPDH (A), p-Akt (Ser473) normalized to GAPDH (**B**) in protein expression were evaluated; (**C**) The relative mRNA expression of Akt was detected by qRT-PCR; (**D**) Quantitative analyses of the ratio of p-Akt to Akt in protein expression was evaluated. Values were represented as mean ± SD (n = 3, each group). The results were representative of three independent experiments. ^#^ *p* < 0.05, ^##^ *p* < 0.05 vs. normal group; * *p* < 0.05, ** *p* < 0.01 vs. H/R group.

The PI3K signaling pathway is involved in multiple cellular processes, including cell apoptosis, proliferation and survival [39]. Akt is one of the major signaling molecules of PI3K. To explore the mechanisms underlying the cardioprotection of TPAE, we investigated some crucial proteins, such as Akt and p-Akt (Ser473) by Western blot and qRT-PCR (Figure 3A,C). After H/R, the levels of Akt, p-Akt (Ser473) decreased markedly compared with those of the normal group (*p* < 0.01), while the ratio of p-Akt to Akt in H/R group slightly increased because of compensation between the lower increase of the Akt protein level compared to that of p-Akt. TPAE pretreatment resulted in significantly increased expressions of Akt and p-Akt (Ser473), especially the ratio of p-Akt to Akt in TPAE (50 μg/mL) group (2.09 ± 0.42 vs. 1.22 ± 0.54, Figure 3A,B,D). The results suggested that pretreatment with TPAE might activate PI3K/Akt signaling pathway through upregulating the p-Akt and Akt.

### 2.4. Inhibition of PI3K Alleviated the Cardioprotection of TPAE

**Figure 4:**
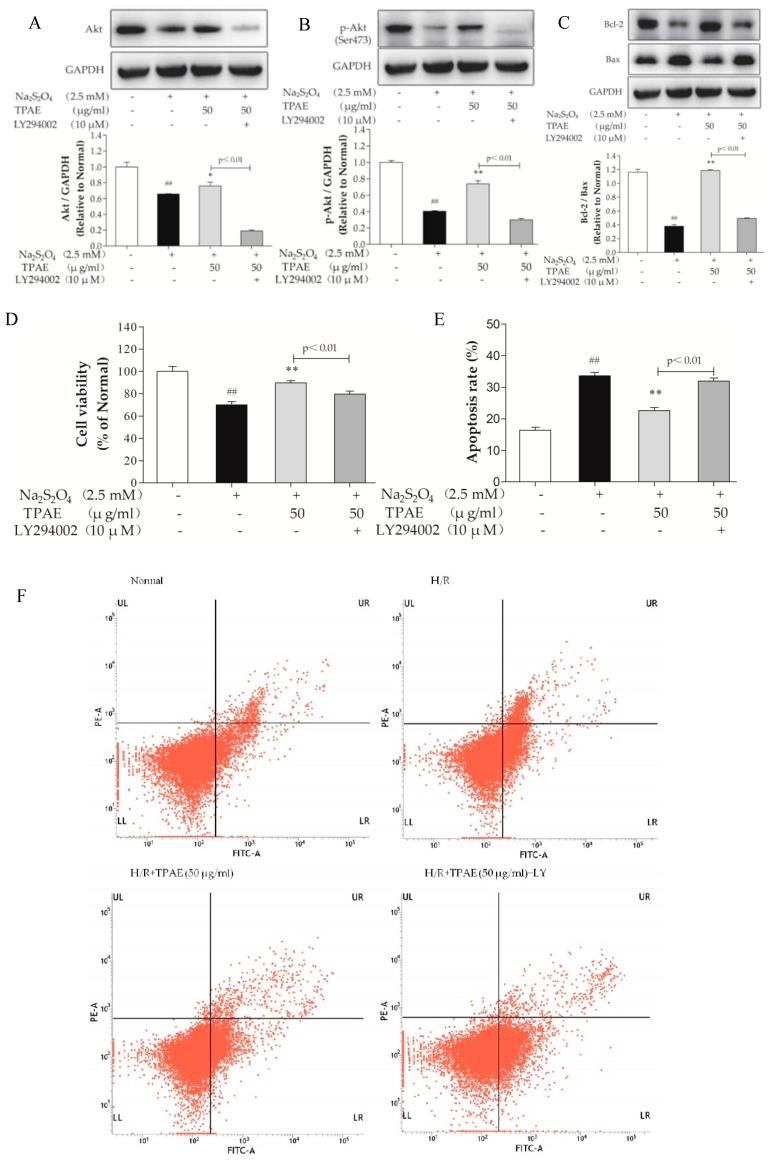
Inhibiting the PI3K/Akt pathway alleviated the cardioprotection of TPAE. H9c2 cells were incubated with 10 μM LY294002 co-treated TPAE (50 μg/mL) for 24 h followed by H/R. The protein expression of Akt (**A**), p-Akt (Ser473) (**B**), Bax (**C**), Bcl-2 (**C**) and GAPDH was measured by western blotting. Quantitative analyses of Akt normalized to GAPDH (**A**), p-Akt (Ser473) normalized to GAPDH (B), the ratio of Bcl-2 to Bax (**C**) in protein expression were evaluated; (**D**) Cell viability was evaluated by MTT assay; (**E**) The apoptosis rate was quantified by BD FACSVerse software; (**F**) The apoptotic ratio of H9c2 cells was measured by flow cytometry using Annexin V-FITC and PI staining. Values were represented as mean ± SD (n = 3 for western blot, n = 6 for MTT, each group). The results were representative of three independent experiments. ^#^ *p* < 0.05, ^##^ *p* < 0.05 vs. normal group; * *p* < 0.05, ** *p* < 0.01 vs. H/R group.

To further confirm the important role of the PI3K/Akt pathway in cardioprotection of TPAE, a specific chemical inhibitor of PI3K, LY294002 was used. Obviously, as shown in Figure 4A, B, the up-regulation of Akt and p-Akt (Ser473) by TPAE was blocked by co-treatment LY294002, and the cell viability significantly decreased (*p* < 0.01, Figure 4D). Furthermore, cell apoptosis and the Bcl-2/Bax expression ratio were correspondingly reversed (Figure 4C,E,F). These results indicated that the PI3K/Akt pathway was involved in the cardioprotective effect of TPAE.

### 2.5. TPAE Mitigated H/R-Induced Decrease of NO Production via PI3K/Akt/NOS Pathway 

**Figure 5:**
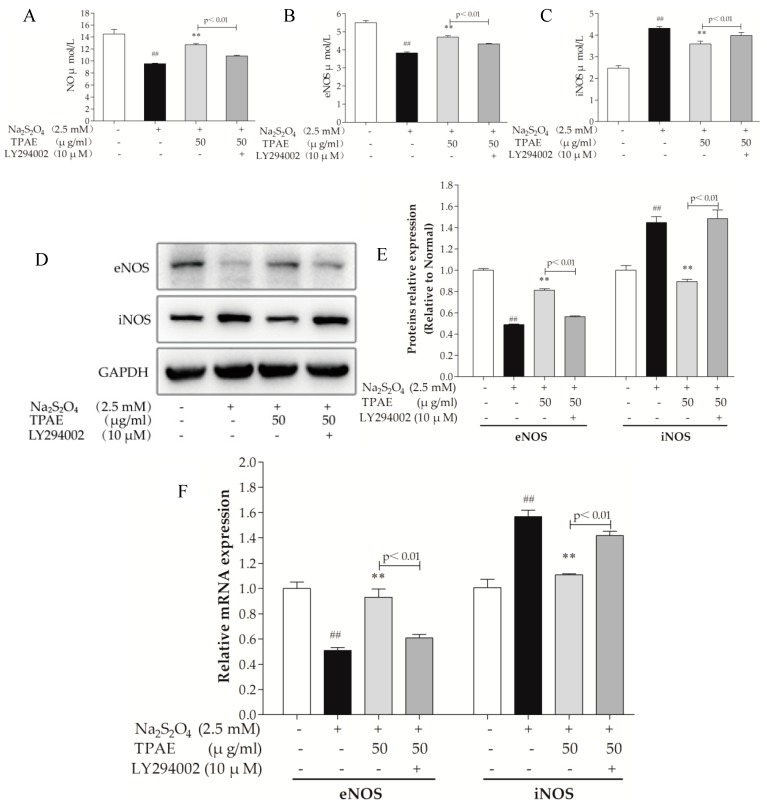
Effects of TPAE on NO, eNOS and iNOS levels. The amount of NO release (**A**), eNOS (**B**) and iNOS (**C**) activities were determined by ELISA kits. (**D**) The protein expression of eNOS, iNOS and GAPDH was measured by western blotting; (**E**) Quantitative analyses of eNOS and iNOS in protein expression were evaluated; (**F**) The relative mRNA expression of eNOS and iNOS was detected by qRT-PCR. Values were represented as mean ± SD (n = 3 for western blot and qRT-PCR, n = 8 for ELISA, each group). The results were representative of three independent experiments. ^#^ *p* < 0.05, ^##^ *p* < 0.05 vs. normal group; * *p* < 0.05, ** *p* < 0.01 vs. H/R group.

Nitric oxide (NO) is generated by a family of NO synthases (NOSs) which catalyze the conversion of the amino acid l-arginine to l-citrulline. It is well known that NO improves cardic cells in the setting of H/R-induced injury via both functional and pathophysiological alterations. Endothelial NOS (eNOS), a downstream enzyme of Akt, is responsible for the production of physiological amounts of NO, while inducible NOS (iNOS) produces NO at high concentrations [40,41]. To determine the effect of TPAE on NO production, we measured the level of NO in the culture medium, examined the intracellular expressions of eNOS and iNOS using ELISA kits, western blot and qRT-PCR analyses. In accordance with the change in the production of NO, the expression of eNOS was obviously downregulated with H/R exposure and significantly upregulated in TPAE pretreated groups (*p* < 0.01, Figure 5A,B). However, compared with the H/R group, the expression of iNOS notably decreased in the TPAE groups (*p* < 0.01, Figure 5C). Our results indicate that recoupling eNOS and reducing iNOS collectively regulate the amount of NO during the H/R. Furthermore, the production of NO, protein expressions of eNOS and iNOS were correspondingly reversed by co-administering with LY294002 (Figure 5A,D–F). All these data clearly demonstrated that TPAE activated NO to protect cardiomyocytes, which could be mediated by PI3K/Akt/NOS pathway. 

## 3. Discussion

Heart failure induced by angina pectoris is a major cause of cardiovascular diseases [5]. TP is a classic traditional Chinese medicine, which has been widely used for curing cardiovascular and cerebrovascular diseases for 2000 years [12,13]. Because of its cardioprotection, reasonable price and safety, it is frequently used for CHD, angina and acute myocardial infarction (AMI) patients in China [42]. In the current study, we showed that TP has beneficial effects in the MI/RI through increasing NO release. Moreover, TP attenuated apoptosis in H9c2 cardiomyocytes in response to H/R. Furthermore, it was confirmed that TP not only activated p-Akt (Ser473), but increased eNOS-mediated NO production, which could be weakened by a PI3K inhibitor, LY294002. These results corporately suggest that TP protects H9c2 cardiomyocytes from hypoxia/reoxygenation injury by regulating NO via PI3K/AKT signaling pathway. 

LDH, which leaks from cells after plasma membrane disruption, can be used as an indicator of cardiomyocytes injury [43]. In this study, we discovered that H/R notably increased LDH leakage compared with the normal group, whereas TP pretreatment significantly blocked LDH production in H/R-induced H9c2 cells (*p* < 0.01, Figure 1C). The observation showed that TP might help protect cardiomyocytes from H/R-induced injury by decreased LDH release. 

Evidence has demonstrated that the apoptosis or death of cardiomyocytes has been implicated in cardiovascular diseases, and the Bcl-2 family proteins are known as vital regulators of the apoptotic response [44,45]. Given that TPAE alleviated H/R-induced injury, we speculated that it consequently reduced apoptosis of H9c2 cells. In the present study, TPAE inhibited apoptosis in H9c2 cardiomyocytes subjected to H/R as evidenced by flow cytometry using Annexin V/PI staining (Figure 2A,B). Additionally, pretreatment of TPAE upregulated the expression of anti-apoptotic Bcl-2 protein and downregulated the production of pro-apoptotic Bax protein in H9c2 cells, resulting in a marked decrease in the Bax to Bcl-2 ratio (*p* < 0.01, Figure 2C–E). Virtually, the ratio between the Bax and Bcl-2 helps to determine the susceptibility of cells to a death signal, and it has been suggested that the Bax to Bcl-2 ratio may be more important than either promoter alone, in determining the apoptosis pathway [46,47]. Thus, these results confirmed that TPAE could regulate the ratio of Bax to Bcl-2 and improve apoptosis of cardiomyocytes.

The PI3K signaling pathway is an essential signaling cascade that promotes cell survival [48]. Previous study has reported that the activation of PI3K signaling pathway can suppress cardiomyocyte apoptosis and promote cell survival in H/R cardiomyocytes [17]. As the major signaling molecule of PI3K and an upstream enzyme of eNOS, Akt is responsible for the production of physiological amounts of cardioprotective NO, while the expression of iNOS and the production of large quantities of NO may contribute to the damage of cardiomyocytes. When PI3K is phosphate-activated by the stimulation of extracellular signal molecules, Akt conformation changes into p-Akt (Ser473) then influences its downstream substrates, which promotes cell survival and inhibits apoptosis. NO is an important signaling molecule that protects cardiac cells in the setting of hypoxia via both functional and pathophysiological alterations. In our current study, the phosphorylation level of Akt was 0.40-fold in the H/R group, respectively, compared with the normal group (*p* < 0.01, Figure 4B). Pretreatment with TPAE prevented the H/R-induced inhibition of Akt phosphorylation. Therefore, we speculated that TPAE could stimulate PI3K/Akt signaling pathway.

The p-Akt (Ser473) and eNOS levels in H9c2 cells pretreated with TPAE were further elevated, following a subsequent increase in NO production, while iNOS expression was inhibited, suggesting that cardioprotective effects of TPAE partly depended on PI3K-Akt-NOS (Figure 5A,D). Furthermore, co-administration of the PI3K inhibitor LY294002 with TPAE pretreatment not only reduced the expression of p-Akt (Ser473) but also markedly decreased eNOS expression, NO production and the Bcl-2/Bax ratio, while iNOS expression was enhanced, which suggested that the presence of iNOS and eNOS was imbalanced after LY294002 stimulation. eNOS protein was upregulated in cardiomyocytes pretreated by TPAE at the stage of H/R, whereas iNOS protein is downregulated, which mainly contributed to the TPAE-induced cardioprotection and anti-apoptotic effect against H/R injury in H9C2 cells (Figure 4B,C; Figure 5A–F). Above all, cardioprotective effects of TPAE were mainly related to recoupling eNOS and reducing iNOS via PI3K/Akt/NO signaling pathway. 

## 4. Materials and Methods 

### 4.1. Materials and Chemicals

Cell culture products were purchased from GIBO BRL Life Technologies (New York, NY, USA). LDH, NO, eNOS, and iNOS RAT ELISA Kits was obtained from LianShuo Biotechnology Co., Ltd (Shanghai, China). Mammalian Cell Lysis Kit and Spin Column Animal total RNA Purification Kit were obtained from Sangon Biological Engineering Technology & Service CO., Ltd (Shanghai, China). Dimethyl sulfoxide (DMSO) and 3-(4, 5-Dimethylthylthiazol-2-yl)-2, 5 diphenylterazolium bromide (MTT) were from Sigma-Aldrich Co. (St. Louis, MO, USA). LY294002 was obtained from ApexBio Technology LLC (Houston, TX, USA). ProtoScript^®^ II First Strand cDNA Synthesis Kit was purchased from New England Biolabs Inc. (Beverly, MA, USA). Applied Biosystems^TM^ PowerUp^TM^ SYBR^®^ Green Master Mix Kit was obtained from Thermo Fisher (Rochford, MI, USA). Primary antibodies against Akt, p-Akt (Ser473), eNOS, iNOS, Bax and Bcl-2 were form Cell Signaling Technology Inc. (Danvers, MA, USA). BCA Protein Assay Kit, Cytoplasmic Protein Extraction Kit, and horseradish peroxidase-conjugated secondary antibodies were obtained from Beyotime Biotechnology Inc. (Beijing, China).

### 4.2. Extract Preparation

The Chinese herbal medicine TP was purchased from the Chinese Medicinal Material Markets (Anguo City, Hebei Province, China), and was identified and assessed by Herbal Identification Staff Room, College of Pharmacy, Liaoning University of Traditional Chinese Medicine. The TPAE was prepared as follows: TP was crushed into fine powder and air dried. Then, 100.0 g of the material was boiled thrice in 1.0 L of distilled water for 2 h. The combined extracts were filtered and pooled, freeze dried and stored at 4 °C until used [12,14].

### 4.3. Cell Culture and Hypoxia/Reoxygenation Model (H/R)

H9c2 rat derived cardiomyocytes (Zishi Biotechnology Co., Ltd., Shanghai, China) were cultured in high glucose DMEM media supplemented with 10% (*v*/*v*) heat-inactivated fetal bovine serum (FBS), including penicillin (100 U/mL) and streptomycin (100 μg/mL). Cells were seeded in a humidified atmosphere containing 5% CO_2_ at 37 °C [39,49]. For all experiments, cells were plated at an appropriate density according to the experimental design and grown to reach 80–90% confluence before experimentations. Before experimental intervention, confluent-cultured cells were serum-starved for 24 h in DMEM supplemented with 0.1% fetal bovine serum [50]. 

Sodium hydrosulfite (Na_2_S_2_O_4_, jkchemical) was applied as a chemical hypoxia inducer [33,35]. Cells were stimulated with 2.5 mM Na_2_S_2_O_4_ (dissolved in phosphate-buffered saline) for 30 min to induce hypoxic condition in vitro and then removed to the regular incubator for 4 h of reoxygenation with the medium replaced by high glucose medium to mimic reperfusion [36,51]. TPAE was firstly dissolved with high glucose DMEM to 2.0 mg/mL, and then diluted with high glucose DMEM to different concentrations (12.5, 25, 50, 100, 200, 400 μg/mL).

### 4.4. Experimental Protocols 

The cultured H9c2 cardiomyocytes were randomly divided into different groups. In the normal group, H9c2 cardiomyocytes were incubated under normal air conditions for equivalent durations with high glucose DMEM. The H/R group was conducted as described in the preceding section. In the H/R + TPAE group, TAPE with three dosages (25, 50, 100 μg/mL) was added to the cultures 24 h before H/R exposure. The H/R + Quercetin group was pretreated with quercetin (25 μM, positive group) for 24 h before hypoxia. In the LY294002 group, cells were incubated with 10μM LY294002 co-administering TPAE for 24 h before treatment with H/R. After 4 h of reoxygenation, cell viability was determined by MTT assay. In the cytotoxic test, cells were treated with various concentrations (12.5, 25, 50, 100, 200, 400 μg /mL) of TPAE for 24 h [52,53]. Then, the cytotoxicity of TAPE was assayed by MTT. The absorbance was measured at 570 nm using microplate reader.

### 4.5. Flow Cytometric Detection of Apoptosis 

To establish the H/R injury model in vitro, the H9c2 cardiomyocytes underwent hypoxia for 30 min followed by reoxygenation for 4 h. The validity of the model in vitro was detected by flow cytometry Annexin V-FITC/PE double staining. After treatment, the samples were collected and washed twice with cold PBS, and then incubated with 5 μL FITC-Annexin V (stained apoptotic cells) and 1 μL PE (stained necrotic cells) working solution (100 μg/mL) for 15 min in the dark at 25 °C. A minimum of 10,000 cells were maintained for all the samples [54,55]. The samples were analyzed by BD FACSVerse^TM^ (NJ, USA).

### 4.6. Enzyme-Linked Immunosorbent Assay

H9c2 cells were cultured in six plates. After experimental procedures, the supernatant was used to measure the cell level of LDH and NO using LDH and NO assay kits. The cells were collected, ultrasonicated, and centrifuged at the speed of 3000 r/min for 5 min at 4 °C. The supernatant was used to assess the activities of iNOS and eNOS according to the corresponding ELISA kits. The cell level of LDH, NO, iNOS and eNOS was expressed as ng/mL, μmol/L, μmol/L and μmol/L. 

### 4.7. RNA Isolation and Purification

The total RNA was extracted and purified from H9c2 cells using Spin Column Animal total RNA Purification Kit. The total RNA concentrations were determined by measuring the optical density at 260 and 280 nm using a NanoDrop 2000 ultramicrospectrophotometer (Thermo, Waltham, MA, USA). According to the gene sequence published in the GeneBank database, primers were designed with Primer 5.0 design software (Table 1). Aliquots of 6 μL RNA from each group were applied for production of cDNA [24,56,57]. The cDNA was synthesized from purified RNA using Applied Biosystems Gene Amp^®^ PCR System 9700 (Rochford, MI, USA), according to the manufacturer’s instructions.

### 4.8. Quantitative Real-Time PCR to Confirm Markers

cDNA was synthesized from 1μg RNA using ProtoScript^®^ II First Strand cDNA Synthesis Kit, according to manufacturer’s instructions. An Agilent Technologies Stratagene Mx 3000p was used for qRT-PCR. Thereafter, the PCR reactions were prepared by adding 10 μL Power Up^TM^ SYBR^TM^ Green Master Mix, 1 μL forward and reverse primers (Akt, iNOS, eNOS, Bax, Bcl-2), 4 μL of cDNA, 0.3 μL ROX Reference Dye, and 4.7 μL RNase-free water to final volume of 20 μL. The qRT-PCR protocol was conducted as follows: 50 °C for 2 min and 95 °C for 5 min, followed by 45 cycles of 95 °C for 20 s, 59 °C for 20 s, 72 °C for 30 s, and then 95 °C for 1 min, 57 °C for 30 s, 95 °C for 30 s [57,58,59]. Each group contained one housekeeping gene, glyceraldehydes-3-phosphate dehydrogenase (GAPDH) against which the sample data were normalized. The reliability of the PCR results was evaluated by the dissolution curve. The cycle threshold (Ct) value was detected and the relative expression of the target gene was calculated according to 2^−ΔΔCt^ method [21]. 

### 4.9. Western Blotting Analysis

After treatment with indicated agents, H9c2 cells were harvested, washed twice with cold PBS, and kept in RIPA lysis buffer for 30 min on ice [1]. Then, the lysates were centrifuged, and the supernatants were collected. Subsequently, the bicinchoninic acid (BCA) was employed to examine the protein concentration. An equivalent amount of protein (24 μg) was resolved by sodium dodecyl sulfate-polyacrylaminde gel electrophoresis (SDS-PAGE) (8–12%) and transferred to polyvinylidene fluoride (PVDF) membranes in Tris-glycine buffer at 400 mA for 40 min [39,60]. The membranes were blocked for 30 min at room temperature with 5% bovine serum albumin (BSA) in Tris-buffered saline containing 0.05% (*v*/*v*) Tween-20 (TBST), and then these membranes were incubated with the primary antibodies (Akt, p-Akt, iNOS, eNOS, Bax, Bcl-2, GAPDH) overnight at 4 °C. After washing three times with TBST, the membranes were incubated for 2 h at room temperature with horseradish peroxidase-conjugated goat anti-rabbit or anti-mouse IgG [61]. Finally, the membranes were washed in TBST, and the protein bands were detected by enhanced chemiluminescence reagents and quantified by densitometry with automatic analysis system. 

### 4.10. Statistical Analysis

All continuous variables are presented as the mean ± standard deviation (SD) of at least three independent experiments. Statistical analysis was performed using GraphPad Prism software version 5.0. Total variation present in a set of data was estimated by one-way analysis of variance (ANOVA) followed by Dunnet’s-test. *p* values < 0.05 were considered statistically significant. 

## 5. Conclusions

**Figure 6:**
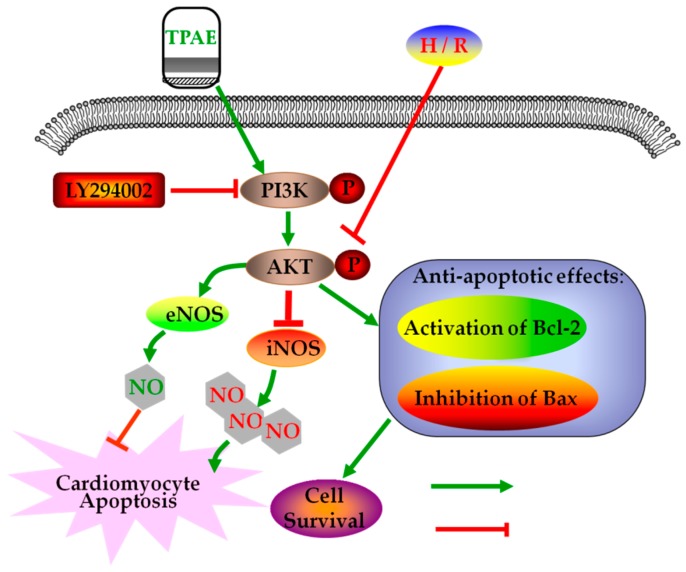
Schematic diagram of the mechanism by which TPAE attenuates H/R induced cardiomyocyte apoptosis by regulating NO production via activating the PI3K/Akt signaling pathway.

In summary, after H/R, we observed the downregulation of anti-apoptotic Akt, p-Akt, Bcl-2, NO production, eNOS level, and upregulation of pro-apoptotic Bax, LDH release, iNOS level. However, pretreatment with TPAE reversed all of the H/R induced effects. Thus, this study provides a cellular and molecular basis for Trichosanthis Pericarpium in the pretreatment of hypoxia/reoxygenation induced injury, at least in part, through PI3K/Akt/NO pathway (Figure 6). Although the protective effects of TPAE have been confirmed in cell experiments, it will be a long period before TPAE can be successfully developed into drugs and applied in clinic. 

## Figures and Tables

**Table 1:** Rat gene-specific primers.

Gene Symbol	Reference Sequence	Primer	Sequence(5′-3′)	Product Size (bp)
Akt	NM_033230.2	Forward	TCACCTCTGAGACCGACACC	110
		Reverse	CCGTTCACTGTCCACACACTC	
eNOS	NM_021838.2	Forward	GGGATTCTGGCAAGACCGAT	151
		Reverse	TTGTCCAAACACTCCACGCT	
iNOS	NM_012611.3	Forward	TTCCTCAGGCTTGGGTCTTGT	213
		Reverse	ATCCTGTGTTGTTGGGCTGG	
Bcl-2	NM_016993.1	Forward	GGATCCAGGATAACGGAGGC	141
		Reverse	ATGCACCCAGAGTGATGCAG	
Bax	NM_017059.2	Forward	GGGCCTTTTTGCTACAGGGT	106
		Reverse	TTCTTGGTGGATGCGTCCTG	
GAPDH	NM_017008.3	Forward	GGCACAGTCAAGGCTGAGAATG	143
		Reverse	ATGGTGGTGAAGACGCCAGTA	

## Supplementary Materials

The following are available online at http://www.mdpi.com/1420-3049/23/10/2409/s1. Table S1. The analytical condition for gradient elution of mobile phases, Table S2. Interactive similarities of TP from different origins, Table S3. Contents of total flavones in TP from different origins, Figure S1. The chromatogram of TP samples and the generated reference standard fingerprint.
