# Supplementary material for: Trichosanthis Pericarpium Aqueous Extract Protects H9c2 Cardiomyocytes from Hypoxia/Reoxygenation Injury by Regulating PI3K/Akt/NO Pathway
Source: Molecules. 2018 Sep 20;23(10):2409. doi: 10.3390/molecules23102409 (PMC6222483; doi:10.3390/molecules23102409)
Supplement: Supplementary file 1 [file molecules-23-02409-s001.zip › molecules-355314-supplementary.pdf]

molecules

Supplementary Materials

Trichosanthis Pericarpium Aqueous Extract Protects H9c2 Cardiomyocytes from Hypoxia/Reoxygenation Injury by Regulating PI3K/Akt/NO Pathway

Donghai Chu [1, 2], and Zhenqiu Zhang [1,*]

[1] Liaoning University of Traditional Chinese Medicine, NO. 77 Shengming 1st Road, Dalian 11600, Liaoning, China; chubotany@sina.cn (D.C.)

[2] Liaoning Institute of Science and Technology, NO.76 Xianghuai Road, Benxi 117004, Liaoning, China

* Correspondence: zhangzhenqiu@sina.com; Tel.: +86-411-8589-0199

Content:

Table S1 The analytical condition for gradient elution of mobile phases. 2

Table S2 Interactive similarities of TP from different origins. 2

Figure S1. The chromatogram of TP samples and the generated reference standard fingerprint. 3

Table S3 Contents of total flavones in TP from different origins. 3

Table S1 The analytical condition for gradient elution of mobile phases.

Time (min)	Mobile phase A	Mobile phase B
0	13.5	86.5
30	16.5	83.5
40	19.5	80.5
50	23.0	77.0
60	35.0	65.0
80	40.0	60.0

Notes: Mobile phase A: methanol mixed with acetonitrile at a volume ratio of 1:10; Mobile phase B: 0.1% phosphate aqueous solution. HPLC fingerprints were performed on an Agilent 1100 series HPLC system. The chromatographic separation was carried out on a Cosmosil MS-II C18 column (250 mm×4.6 mm, 5 μm), operated at 25°C. The analytical condition was set as shown in Table 1. The flow rate was 1.0 mL / min and the detection wavelength was set at 360 nm with the sample injection volume of 20 μL.

Table S2 Interactive similarities of TP from different origins.

Sample number	S_1	S_2	S_3	S_4	S_5	S_6	S_7	S_8	Standard fingerprint
S_1	1	0.97	0.993	0.956	0.974	0.987	0.981	0.959	0.996
S_2	0.97	1	0.967	0.926	0.949	0.965	0.958	0.942	0.974
S_3	0.993	0.967	1	0.933	0.971	0.996	0.991	0.98	0.997
S_4	0.956	0.926	0.933	1	0.925	0.91	0.912	0.882	0.941
S_5	0.974	0.949	0.971	0.925	1	0.973	0.975	0.954	0.978
S_6	0.987	0.965	0.996	0.91	0.973	1	0.993	0.982	0.995
S_7	0.981	0.958	0.991	0.912	0.975	0.993	1	0.983	0.992
S_8	0.959	0.942	0.98	0.882	0.954	0.982	0.983	1	0.975
Standard fingerprint	0.996	0.974	0.997	0.941	0.978	0.995	0.992	0.975	1

Notes: (S_1: Hebei Province, S_2: Sichuan Province, S_3: Guizhou Province, S_4: Yunnan Province, S_5: Henan Province, S_6: Shandong Province, S_7: Shanxi Province, S_8: Anhui Province)

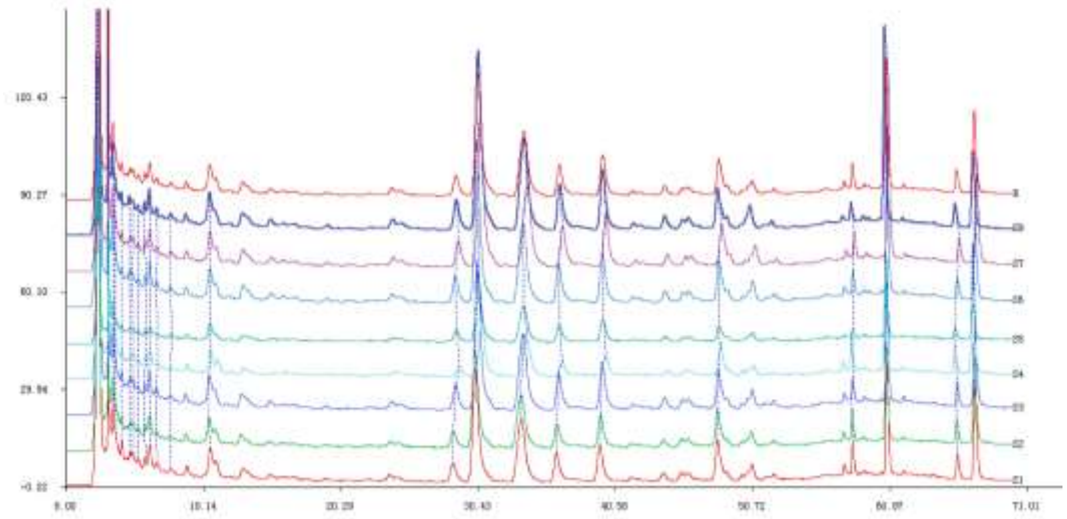

Figure S1. The chromatogram of TP samples and the generated reference standard fingerprint.

Note: S_1-S_8: fingerprints of 8 batches of TP samples from different origins; R: reference standard fingerprint created by Similarity evaluation system for chromatographic fingerprint of traditional Chinese Medicine, version 2004 A.

Table S3 Contents of total flavones in TP from different origins.

Sample number	Contents of total flavones(mg/g)	Mean (mg/g)	RSD
S_1	5.829	6.128	2.96%
S_2	5.989		
S_3	6.014		
S_4	6.126		
S_5	6.296		
S_6	6.365		
S_7	6.125		
S_8	6.282		
